# Studies on Sorption and Release of Doxycycline Hydrochloride from Zwitterionic Microparticles with Carboxybetaine Moieties

**DOI:** 10.3390/ijms25147871

**Published:** 2024-07-18

**Authors:** Stefania Racovita, Marin-Aurel Trofin, Ana-Lavinia Vasiliu, Mihaela Avadanei, Diana Felicia Loghin, Marcela Mihai, Silvia Vasiliu

**Affiliations:** “Petru Poni” Institute of Macromolecular Chemistry, 41 A Grigore Ghica Voda Alley, 700487 Iasi, Romania; marin.trofin@icmpp.ro (M.-A.T.); vasiliu.lavinia@icmpp.ro (A.-L.V.); mavadanei@icmpp.ro (M.A.); diana.loghin@icmpp.ro (D.F.L.); marcela.mihai@icmpp.ro (M.M.); silvia.vasiliu@icmpp.ro (S.V.)

**Keywords:** zwitterionic microparticles, doxycycline hydrochloride, sorption kinetics, sorption isotherms, drug delivery systems

## Abstract

The aim of this study was to examine the use of zwitterionic microparticles as new and efficient macromolecular supports for the sorption of an antibiotic (doxycycline hydrochloride, DCH) from aqueous solution. The effect of relevant process parameters of sorption, like dosage of microparticles, pH value, contact time, the initial concentration of drug and temperature, was evaluated to obtain the optimal experimental conditions. The sorption kinetics were investigated using Lagergren, Ho, Elovich and Weber–Morris models, respectively. The sorption efficiency was characterized by applying the Langmuir, Freundlich and Dubinin–Radushkevich isotherm models. The calculated thermodynamic parameters (ΔH, ΔS and ΔG) show that the sorption of doxycycline hydrochloride onto zwitterionic microparticles is endothermic, spontaneous and favorable at higher temperatures. The maximum identified sorption capacity value is 157.860 mg/g at 308 K. The Higuchi, Korsmeyer–Peppas, Baker–Lonsdale and Kopcha models are used to describe the release studies. In vitro release studies show that the release mechanism of doxycycline hydrochloride from zwitterionic microparticles is predominantly anomalous or non-Fickian diffusion. This study could provide the opportunity to expand the use of these new zwitterionic structures in medicine and water purification.

## 1. Introduction

A healthy population is fundamental to the progress of any society. In order to achieve this objective, there is a continuous interest in the prevention and treatment of diseases. Thus, many research studies are directed towards obtaining drug delivery systems. The aim of these systems is to improve efficacy by controlling the rate, time and location of drug release in the body. A material, especially a polymeric material, is a good candidate for the preparation of drug delivery systems if it fulfills some important properties: biocompatibility, low toxicity, immunogenicity, ability to load with an effective amount of drug and, of course, the ability to deliver the drug to the target. At the same time, the interaction between the polymer and the blood components must be minimal. In order to keep the drug’s half-life as long as possible, its removal should be avoided; otherwise, it may be inactivated before reaching the target cells [1].

In recent years, zwitterionic polymers have represented an important research direction in the development of new advanced biomaterials due to their antifouling and antimicrobial properties, easy functionalization and flexibility of structural design [2]. According to the distribution of ionic groups within the macromolecular chain, zwitterionic polymers are divided into two categories [3]: (i) polyampholytes—polymers that have the positive and negative ionic groups located on different structural units [4]; (ii) polybetaines—polymers that have the cationic and anionic groups in the same repeating unit, separated by an alkyl chain with variable length [5]. The cationic groups presented in the structure of polybetaines are represented by the quaternary ammonium groups. Depending on the anionic group (carboxylate, sulphonate, phosphate), they are classified into poly(carboxybetaine), poly(sulfobetaine) and poly(phosphobetaine).

A number of studies have focused on the use of poly(carboxybetaines) as drug delivery systems. The studies of our working group were directed towards the synthesis and use of linear and crosslinked polybetaines in the development of systems with controlled/sustained drug release. Starting from chitosan and two linear poly(carboxybetaines) based on N-vinylimidazole with one and two methylene groups between ionic groups, we obtained core–shell microparticles, which were subsequently tested for the adsorption and release of the antibiotic chloramphenicol hemisuccinate sodium salt [6]. Another oral delivery system consisting of graft copolymers with a carboxybetaine structure, based on gellan and N-vinylimidazole [7], was used to immobilize two antibiotics: cefotaxime sodium salts and triazole (3-(benzoxazole-2′-yl-mercapto-methyl)-4-(p-methoxyphenyl)-5-mercapto-1,2,4-triazole) [8]. Gu and collaborators [9] directed their research towards the preparation of ternary nanoparticles based on poly(carboxybetaine methacrylate), peptide dendrimer-modified carbon dots and doxorubicin that have been used in cancer therapy. Poly (carboxybetaine)/tetraethylene glycol diacrylate prepared by the water-in-oil emulsion technique shows excellent hydrolytic degradability, with this property being tailored by changing the crosslinking density [10].

Currently, sulfobetaine polymers are widely studied as drug delivery systems due to their properties: they possess biomimetic motifs, are stimuli-responsive polymers and exhibit an upper critical solution temperature. Poly(sulfobetaine methacrylate) [poly(SBMA)] has a similar structure to taurine, a non-essential sulfur-containing amino acid that is found in the human body. Therefore, a combination of poly(SBMA) and other synthetic or natural polymers may lead to interesting materials with important biological properties (biocompatibility, antifouling capacity, biodegradability, hemocompatibility) [11,12]. For example, sulfobetaine-functionalized polyacetal dendrimers were used as an antitumor drug delivery system [13], while biodegradable nanogels based on poly(sulfobetaine methacrylate) loaded with doxorubicin exhibited the strongest growth inhibition effect on hypopharyngeal carcinoma [14].

Along with poly(carboxybetaines) and poly(sulfobetaines), poly(phosphobetaiens) containing a phosphorylcholine group represent other important members of the polybetaine family that have been evaluated as drug delivery systems [15]. Thus, poly(2-methacryloyloxyethyl phosphorylcholine) (PMPC), alone or in combination with other polymers, was used to prepare various drug delivery systems such as nanogels sensitive to low oxygen concentrations for chemotherapy of glioblastoma [16], polymer–drug conjugates used in malignant tumor therapy [17,18,19], vesicles [20] and biomimetic micelles [21] for the encapsulation of anticancer drugs (doxorubicin and paclitaxel). Also, PMPC was used to modify the poly(amidoamine) dendrimer surface to improve drug delivery capacity to brain tumors, as well as to simultaneously reduce the toxicity of dendrimers and the tissue toxicity of loaded doxorubicin [22].

This work highlights the preparation of a novel drug delivery system based on crosslinked polymers with betaine moieties and DCH. Crosslinked polymers with betaine moieties in the form of porous microparticles are less studied in the literature, and the interaction between these types of macromolecular supports and DCH, as well as the release mechanism of the drug, represents an interesting field of study that can be a starting point in the further development of synthetic zwitterionic materials with potential applications in the biomedical field, especially as oral drug delivery systems in the treatment of the infectious diseases. The DCH, used as a model drug, is an antibiotic of the family of tetracycline which is efficacious against most Gram-negative and Gram-positive bacteria [23,24]. This antibiotic is used to treat intestinal, urinary and respiratory infections in humans [24]. Since the drug was immobilized into a polymer matrix by sorption, the influence of several parameters (dosage of microparticles, pH value, contact time, initial concentration of drug and temperature) was studied to find the optimal immobilization conditions.

## 2. Results and Discussion

The precursor microparticles (P microparticles) were obtained by a simple polymerization method, namely the suspension polymerization method [25], while the zwitterionic microparticles (PB_1_, PB_2_ and PB_3_ microparticles) were prepared by the betainization reaction at tertiary nitrogen from the structure of P microparticles with sodium monochloracetate, acrylic acid and methacrylic acid, respectively. The preparation of these microparticles was presented in our previous study [26]. The main characteristics of the precursor and zwitterionic microparticles are shown in Table 1.

### 2.1. Selection of Key Factors for Sorption of Doxycycline Hydrochloride onto Precursor and Zwitterionic Microparticles

**Figure 1:**
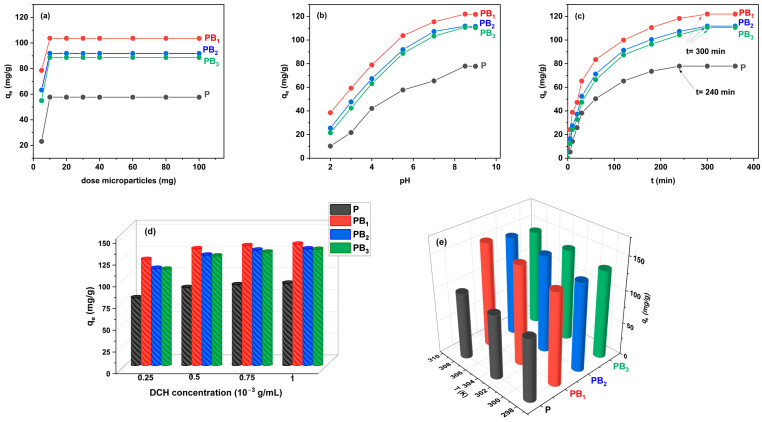
The effect of factors on the DCH sorption process on precursor/zwitterionic microparticles: (**a**) dosage of microparticles, (**b**) pH value, (**c**) contact time, (**d**) initial concentration of drug and (**e**) temperature.

The influence of key factors (dosage of microparticles, pH value, contact time, the initial concentration of drug and temperature) on the DCH sorption capacity of precursor and zwitterionic microparticles was investigated in order to find the optimal conditions for drug loading and is presented in Figure 1.

#### 2.1.1. Effect of the Dose of Precursor and Zwitterionic Microparticles on Sorption of DCH

The influence of the dose of precursor and zwitterionic microparticles on the amount of sorbed DCH (Figure 1a) was achieved by keeping the other parameters constant (C_DCH_ = 0.25 × 10^−3^ g/mL, pH = 5.5, t = 420 min, T = 298 K). From Figure 1a, it can be seen that at a dose of microparticles higher than 10 mg, the amount of DCH remains constant, with equilibrium being established between the concentration of DCH on the sorbent surface and in the bulk solution. Due to the fact that the dose of microparticles ≥ 10 mg, the amount of sorbed drug mainly depended on the solution concentration of DCH and less on the amount of microparticles. Furthermore, a high dose of microparticles can be disadvantageous because their agglomeration can occur and thus reduce the sorption performance per mg of microparticles. It is also observed that the optimal dose is the same for both precursor and zwitterionic microparticles, namely 10 mg.

#### 2.1.2. Effect of pH Solution

It is well known that the pH of a drug solution is an important factor that affects the sorption capacities of sorbents [27]. The pH of a drug solution influences both the dissociation and ionization degree as well as the surface charge of the sorbents and, as a consequence, has an influence on the sorbent–sorbate interactions [28]. For this reason, the influence of pH on the sorption performance of precursor and zwitterionic microparticles for DCH was studied. Figure 1b shows the influence of pH values on the sorption capacity of DCH onto P, PB_1_, PB_2_ and PB_3_ microparticles in the following conditions: DCH concentration of 0.25 × 10^−3^ g/mL, dose of microparticles of 10 mg, contact time of 420 min and temperature of 298 K.

It is evident that the DCH sorption on the precursor and zwitterionic microparticles’ surfaces was highly pH-dependent. Moreover, DCH can act as an anion, cation or zwitterion and therefore possesses several values of the acid dissociation constant (pK_a1_ = 3.02, pK_a2_ = 7.97, pK_a3_ = 9.15). Thus, when the pH < 3.02, DCH presents positive charges, acts as a zwitterion at pH values between 3.02 and 7.97, and is negatively charged at pH > 7.97 DCH [29]. The amount of drug sorbed increased with the increase in the pH value of the drug solution, and the highest sorption performance was obtained at pH 8.5. The presence of carboxybetaine units in the zwitterionic microparticle structure plays a significant role in the sorption process, leading to higher drug sorption than in the case of precursor microparticles. The maximum sorption at pH = 8.5 was probably governed both by the electrostatic interactions between the polymeric matrix and the drug, as well as by π-π interactions between the aromatic ring of the DCH and the imidazole ring in the microparticle structures. The highest amount of DCH sorbed was observed for PB_1_ microparticles, probably due to their higher degree of betainization as well as the higher swelling degree as compared to the other zwitterionic microparticles (Table 1).

**Figure 2:**
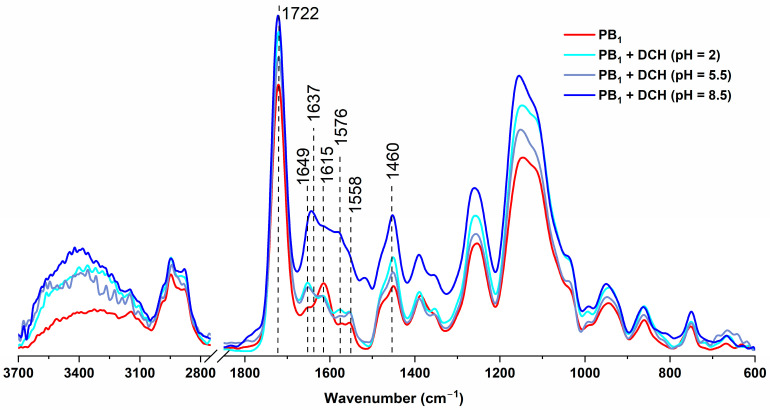
ATR-FTIR spectra of PB_1_ microparticles before and after DCH loading at different pH values.

DCH sorption onto microparticles at several pH values was highlighted by ATR-FTIR spectroscopy (Figure 2).

The presence of DCH brings changes in the ATR-FTIR spectra as compared to the spectrum of the initial microparticles, mainly in the area of 1680–1530 cm^−1^, presenting the following specific bands:

- 1649 and 1637 cm^−1^: absorption bands attributed to the amide I groups and the C=O ketone bond from DCH;
- 1615 cm^−1^: absorption band belonging to the phenol group in the DCH structure overlapping the ν_asym_(COO^−^) band of betaine and the specific ν(C=C) band;
- 1576 cm^−1^: low-intensity absorption band that is specific to the ν(C=C) bond in the aromatic cycle belonging to DCH. This band appears for all types of drug-loaded zwitterionic microparticles at the same wavenumber;
- 1558 cm^−1^: a band specific to the amide II group in DCH, which is very visible for the PB_1_+DCH microparticles loaded at pH 8.5 and does not appear in the case of systems where sorption took place at lower pH values (2 and 5.5);
- 1460 cm^−1^: wide absorption band representing a combination of specific DCH bands, namely ν(C=C) from the aromatic ring, δ(C–OH) and δ(C–CH_3_). This broader band overlaps the specific band of zwitterionic PB_1_ microparticles, leading to an increase in the intensity of the final band. The absorption spectrum of the PB_1_+DCH system shows the presence of a lower-intensity band at 1453 cm^−1^, suggesting the existence of certain types of interactions between the drug and the polymer matrix such as electrostatic interactions and π-π interactions between the aromatic ring from DCH and the imidazole ring belonging to the PB_1_ microparticles.

**Figure 3:**
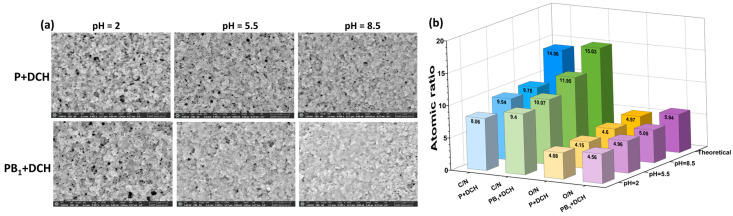
(**a**) SEM images and (**b**) experimental and theoretical atomic ratios of P and PB_1_ microparticles after DCH loading at different pH values.

The surface of P and PB_1_ microparticles loaded with DCH was observed by SEM microscopy to highlight the impact of pH on the morphology (Figure 3a) and elemental composition (Figure 3b).

SEM images suggest that the surface morphologies of precursor and zwitterionic microparticles are influenced by the pH of the DCH solution (Figure 3a). Thus, the surface of microparticles becomes more compact when the pH of the DCH solution increases, leading to the conclusion that more drug is sorbed under these conditions.

EDAX analysis highlights the presence of chemical elements such as carbon, oxygen and nitrogen on the structure of the microparticles + DCH systems. The decreases in the values of C/N and O/N atomic ratios (Figure 3b) in microparticles + DCH systems as compared to those of P and PB_1_ microparticles without the drug confirm the presence of DCH in the structure of precursor/zwitterionic microparticles. In the case of P and PB_1_ microparticles, with the increase in pH, an increase in the values of C/N (from 8.06 to 9.78, and from 9.4 to 11.95, respectively) as well as O/N (from 4.08 to 4.6, and from 4.56 to 5.05, respectively) atomic ratios is observed. The best loading efficiency was observed when the sorption process of DCH was carried out onto PB_1_ microparticles at pH = 8.5. Thus, it can be concluded that the maximum sorption capacity of DCH on precursor and zwitterionic microparticles was obtained at pH 8.5.

#### 2.1.3. Effect of Contact Time

It is known that the contact time influences the sorption kinetics and is therefore another important factor in drug sorption studies. The influence of contact time on the sorption process is plotted in Figure 1c (C_DCH_ = 0.25 × 10^−3^ g/mL, dose of microparticles = 10 mg, pH = 8.5, T = 298 K), showing that the equilibrium is attained in 240 min for precursor microparticles. Also, a higher amount of drug sorbed onto all three types of zwitterionic microparticles was observed compared to the P microparticles, which required a longer period of time until equilibrium was attained. The sorption of DCH is fast in the first 60 min since a large number of binding sites from the microparticles are available for sorption of the drug, and thereafter, it becomes slower until equilibrium is established. At longer contact times, the amount of DCH sorbed remains constant.

#### 2.1.4. Effect of the Initial Concentration of Drug Solution

The effect of the initial concentration of DCH solution on the sorption capacity of precursor and zwitterionic microparticles was studied in the concentration range of 0.25 × 10^−3^–1 × 10^−3^ (g/mL) using 10 mg microparticles (contact time = 360 min, pH = 8.5, T = 298 K). Figure 1d shows that the sorption capacity increases with the increase in the DCH concentration of the initial solution. At a high concentration of the DCH solution, the number of interactions between the sorbent and sorbate ions increases, leading to an increase in the amount of DCH sorbed. A high amount of DCH sorbed was observed for zwitterionic microparticles. This can be explained most probably by the presence of carboxybetaine units that increase the hydrophilicity of microparticles and, in consequence, provide a higher swelling degree, facilitating an easier penetration of DCH into the pores of the microparticles. In the case of zwitterionic microparticles, electrostatic interactions between ionic groups belonging to the drug and ionic groups of carboxybetaine moieties were established. A higher amount of drug was obtained for the initial concentration of drug c_i_ = 1 × 10^−3^ (g/mL).

#### 2.1.5. Effect of Temperature

Temperature is another important parameter to be taken into account in the sorption process of DCH on precursor and zwitterionic microparticles (Figure 1e). Thus, with an increase in the temperature from 298 K to 308 K, the sorption capacities of precursor and zwitterionic microparticles increased as follows: from 94.65 mg/g to 100.5 mg/g (P microparticles); 139.56 mg/g to 157.86 mg/g (PB_1_ microparticles); 134.11 mg/g to 150.4 mg/g (PB_2_ microparticles); and 133.51 mg/g to 144.1 mg/g (PB_3_ microparticles), indicating that the sorption process is endothermic. These results led to the conclusion that the sorption of the drug is favored by an increase in temperature, due to an increase in the diffusion rate that facilitates the transport of the DCH molecules across the surface and into the internal pores of microparticles. Increasing the temperature shows a positive impact on the sorption process, but an increase in the temperature value above 308 K can induce thermal degradation of DCH [30]. Therefore, DCH sorption studies were performed in the range of 298–308 K to prevent degradation of the antibiotic into compounds with lower antimicrobial activity and increased toxicity [31].

Following the above-presented parameters, the optimal DCH sorption conditions for the precursor/zwitterionic microparticles are as follows: dose of microparticles = 10 mg, pH = 8.5, T = 308 K, and initial concentration of DCH solution = 1 × 10^−3^ g/mL.

### 2.2. Sorption Kinetics

**Figure 4:**
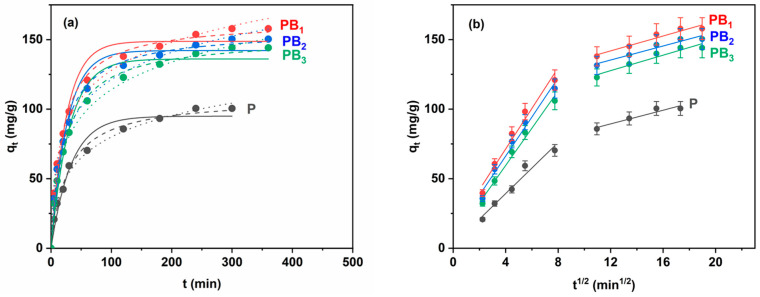
(**a**) Lagergren (solid line), Ho (dash line) and Elovich (dot line) models and (**b**) Weber–Morris model for DCH sorption on the precursor and zwitterionic microparticles (C_DCH_ = 1 × 10^−3^ g/mL, pH = 8.5, and T = 308 K).

The nonlinear fitted results of the Lagergren, Ho and Elovich models for DCH sorption on the precursor and zwitterionic microparticles are shown in Figure 4a. The values of the kinetic parameters and the error functions (R^2^ and χ^2^) are presented in Table 2.

For DCH sorption onto zwitterionic microparticles (Table 2), it is observed that the q_e_ values calculated by applying the Ho model are close to the experimental values as compared to the q_e_ values calculated by applying the Lagergren model. Moreover, the higher values of R^2^ along with low values of χ^2^ suggest that the Ho model better describes the experimental data. It was also observed that in the case of DCH sorption on precursor microparticles, although the value of q_e_ is close to the value of q_e,exp_, the small value of R^2^ (0.970) associated with a higher value of χ^2^ (38.626) shows that the Lagergren model does not describe the experimental data well. These results indicate that the pseudo-second-order model provided an adequate correlation for DCH sorption onto P, PB_1_, PB_2_ and PB_3_ microparticles. As compared to P microparticles, the values of k_2_ for zwitterionic microparticles indicate higher drug loading, which can be correlated with their higher swelling degree (the swelling degree varies in the order PB_1_ > PB_2_ > PB_3_ > P). In addition, in the case of zwitterionic microparticles, the values of the rate constant k_2_ increase with an increase in the betainization degree, suggesting a higher sorption rate of DCH on PB_1_. Also, the high values of R^2^ correlated with low values of χ^2^ obtained in the case of the Elovich model indicate that this model describes the experimental data well. Hence, the application of the Elovich model provides a further argument that the sorption mechanism of DCH on precursor and zwitterionic microparticles is of chemical nature.

It is known that the sorption process can take place in one or more stages [32]. These steps may include film or external diffusion, pore diffusion, surface diffusion and sorption on the pore surface. Because the Lagergren and Ho models cannot accurately identify the diffusion mechanism, in this case, the experimental data were analyzed using the intra-particle diffusion model. The straight-line plots of q_t_ versus t^1/2^ were used to determine the constants k_id_ and I as slope and intercept, respectively (Figure 4b). According to the Weber–Morris equation (see Section 3), if the plot gives a straight line, then the sorption will be controlled exclusively by the intra-particle diffusion, but if multilinear plots are obtained, there are two or more steps that influence the sorption process [32]. The values of k_id_, I_d_ and the correlation coefficients are presented in Table 3.

As can be seen from Figure 4b, the studied sorption process has two straight-line plots. Also, as shown in Table 3, the values of k_id_ are higher for the first straight line as compared to the second straight line (k_i1_ > k_i2_), probably due to the difference between the shape and size of the pores of the microparticles. Therefore, it is plausible to assign the first linear region from Figure 4b to macropore diffusion, whereas the second linear region can be assigned to mesopore diffusion. The I values increase in the following order: P < PB_3_ < PB_2_ < PB_1_ microparticles. These results also confirm that the best sorbent is represented by the PB_1_ microparticles.

### 2.3. Sorption Isotherms

It is important to know the conditions necessary to establish the sorption equilibrium in order to determine whether a polymeric material will be a good sorbent under different experimental conditions. The equilibrium data were fitted with the Langmuir, Freundlich and Dubinin–Radushkevich isotherm models, and the associated isotherm parameters and the correlation coefficients are listed in Table 4 and Table 5.

Based on the analysis of the data from Table 4 and Table 5, the following conclusions can be drawn:

- The values of the maximum sorption capacity (q_m_) calculated on the basis of the Langmuir isotherm are close to the experimental values (q_e,exp_);
- The K_L_ values increase with an increase in temperature, showing a higher DCH sorption efficiency at higher temperatures. Also, the K_L_ value is highest for zwitterionic microparticles and indicates a higher affinity for DCH compared to precursor microparticles, which is in concordance with the sorption capacity obtained from the experimental data;
- For zwitterionic microparticles, the K_L_ values increase with increasing degree of betainization, indicating a higher sorption rate of DCH on PB_1_;
- The high values achieved for R^2^ (0.995–0.999) and small values for χ^2^ (0.070–0.475) suggest good fitting of the sorption data in the case of the Langmuir model;
- The values of 1/n_F_ are in the range of 0–1, which shows that the Freundlich isotherm is favorable in the case of DCH sorption onto P, PB_1_, PB_2_ and PB_3_ microparticles. But the small values of R^2^ (0.926–0.966) along with the high values of χ^2^ (1.083–5.404) suggest that the Freundlich isotherm does not describe the experimental data well;
- The values of the maximum sorbed amount obtained by applying the Dubinin–Radushkevich isotherm show that the q_m_ values are very close to the experimental values, which indicates that this isotherm describes the experimental data well. This is also supported by the fact that the values close to unity for the correlation coefficient R^2^ are associated with low values of χ^2^, confirming that the Dubinin–Radushkevich isotherm describes the sorption of DCH on precursor and zwitterionic microparticles very well;
- In the case of DCH sorption on precursor microparticles, the process is physicochemical (7.030 < E< 9.060 kJ/mol), and in the case of DCH sorption on zwitterionic microparticles, the process takes place through ion exchange (8.003 < E < 11.273 kJ/mol).

### 2.4. Thermodynamic Studies

The corresponding values of thermodynamic parameters (ΔG, ΔH and ΔS) are given in Table 6.

The positive values of ΔH situated in the range of 20–38 kJ/mol indicate that the sorption process is of an endothermic nature and the interaction between precursor/zwitterionic microparticles and DCH is of a physicochemical nature [33]. The negative values of ΔG at all temperatures show the spontaneous nature and the feasibility of the sorption process of DCH onto P, PB_1_, PB_2_ and PB_3_ microparticles. Moreover, the significant decrease in the negativity of the ΔG value with increasing temperature indicates a favorable DCH uptake at higher temperatures [34]. The positive values of ΔS suggested the increase randomness at the solid/solution interface during the sorption of DCH onto P, PB_1_, PB_2_ and PB_3_ microparticles [35].

### 2.5. In Vitro Release Studies

**Figure 5:**
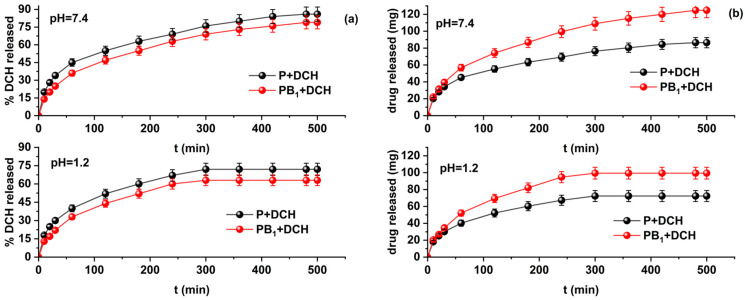
Release profiles of DCH from P+DCH and PB_1_+DCH microparticles represented as (**a**) the percentage and (**b**) the cumulative amount of drug released.

Release studies were performed for DCH-loaded precursor microparticles and DCH-loaded zwitterionic microparticles containing the highest amount of sorbed drug, namely P+DCH and PB_1_+DCH, respectively. The release profiles are represented in Figure 5.

From the graphical representations in Figure 5, it can be seen that the release process of DCH from the precursor microparticles occurs at a higher rate than that for the zwitterionic microparticles. The release of DCH is faster at pH = 1.2 than at pH = 7.4; this phenomenon is explained by the fact that the microparticles present a higher swelling degree at pH = 1.2. At pH values lower than the specific pK_a_ values of carboxylic groups (pK_a_ = 4.5), the zwitterionic microparticles are transformed into the corresponding cationic polyelectrolytes. At pH = 7.4, the carboxylic groups convert to carboxylate groups and the crosslinked structures return to their zwitterionic form with a decrease in the swelling degree. This behavior can be explained by the possibility of the formation of an inner-salt structure by neutralization between N^+^ and COO^−^ in the same betaine unit.

**Figure 6:**
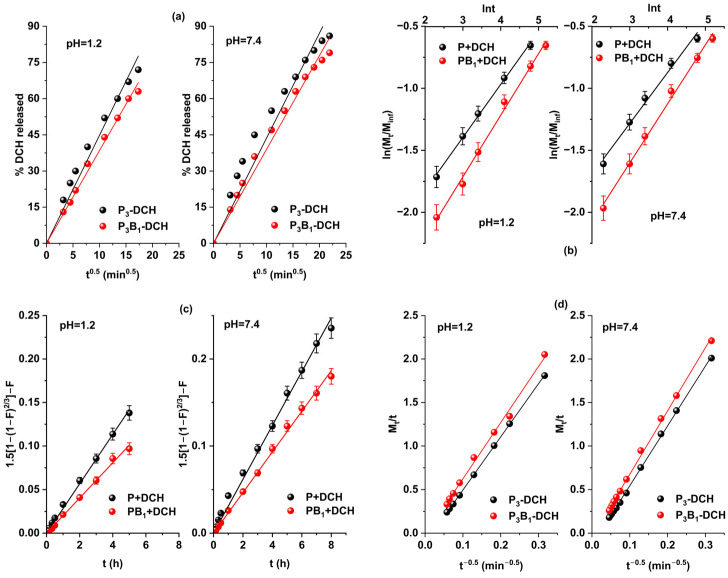
Graphical representations of (**a**) Higuchi, (**b**) Korsmeyer–Peppas, (**c**) Baker–Lonsdale and (**d**) Kopcha mathematical models applied for drug release from P+DCH and PB_1_+DCH microparticles.

The interpretation of the DCH release kinetics of P+DCH and PB_1_+DCH samples was performed using the Higuchi, Korsmeyer–Peppas and Baker–Lonsdale mathematical models. The diffusion and erosion contributions to the release patterns were quantified using the Kopcha model. Figure 6 shows graphical representations of the four mathematical models, and the values of the kinetic parameters are shown in Table 7.

The release data were fitted adequately for all four mathematical models with R^2^ values higher than 0.985. The rate constants obtained by applying the Higuchi, Korsmeyer–Peppas and Baker–Lonsdale kinetic models indicate that the release rate of DCH from the precursor microparticles is higher than that for the zwitterionic microparticles. The difference between the amounts of drug released from precursor and zwitterionic microparticles can be explained by the electrostatic interactions and π-π interaction of DCH with the functional groups belonging to the chemical structure of the zwitterionic microparticles. In the case of precursor microparticles, the drug is retained on the surface of microparticles in a larger amount, which is also the reason for the faster release of the drug.

It is known that the Baker–Lonsdale model is derived from the Higuchi model, and if the graphical representation is a straight line, then the drug release mechanism is controlled by the diffusion phenomenon. From Figure 6c, it can be seen that the graphical representations of the Baker–Lonsdale model are straight lines with very good correlation coefficients (R^2^ ≥ 0.997), which indicates that the release of DCH from P+DCH and PB_1_+DCH microparticles is controlled by the diffusion phenomenon.

Also, the Korsmeyer–Peppas and Kopcha models are two commonly used mathematical models to understand the mechanism of drug release. It is known that the diffusion exponent n in the Korsmeyer–Peppas model gives us information about the release mechanism. In this study, in the case of P+DCH microparticles, the value of n < 0.43 indicates that the release mechanism of DCH is a Fick-type diffusion mechanism. For PB_1_+DCH microparticles, the value of n is in the range of 0.476–0.496, indicating that the release of DCH took place by a complex mechanism, controlled by both diffusion and swelling processes characteristics for anomalous or non-Fickian diffusion. Also, the values of the n diffusion exponent are less than 0.85, leading to the conclusion that the microparticles swelled but did not undergo any disintegration or erosion processes [36].

The Kopcha model is commonly used to indicate the relative contribution of diffusion and erosion processes to drug release [37]. According to the Kopcha model, if A > B, the dominant drug release mechanism is diffusion, while for A < B, then erosion predominates [34]. It is observed that the Kopcha model describes the experimental data very well (R^2^ ≥ 0.995) and A is higher than B (the A/B ratio greater than 1), which confirms that the release mechanism is predominantly based on the diffusion process.

### 2.6. Comparison with Other Sorbents

Comparative sorption studies are important to evaluate the efficacy of a sorbent to a specific sorbate. Therefore, to illustrate the performance of P and PB_1_ microparticles for the sorption of DCH, a comparison with other porous sorbent materials presented in the literature [35,36,37,38] is shown in Table 8.

As can be seen, the sorption capacity of P and PB_1_ microparticles for DCH obtained in this work has comparative values with those of the other porous sorbent materials presented in the literature, with the zwitterionic microparticles showing better sorption capacity than already used mesoporous silica, whereas the precursor microparticles have similar properties to the composite of silica with ZnO.

## 3. Materials and Methods

### 3.1. Materials

All reagents employed in this study were procured from Sigma-Aldrich, Darmstadt, Germany. The monomers glycidyl methacrylate (GMA) and N-vinylimidazole (NVI) and the betainizing agents (acrylic acid and methacrylic acid) used in the preparation of the precursor and zwitterionic microparticles were purified by vacuum distillation before use. Triethylene glycol dimethacrylate (TEGDMA), the initiator [benzoyl peroxide (BOP)], porogenic agent (n–butyl acetate), poly(vinyl alcohol) (PVA, M_w_ = 52,650 g/mol, degree of hydrolysis 88%), gelatin, MgCl_2_·6H_2_O, methanol, sodium monochloracetate (98%), AgNO_3_, HCl, NaOH and DCH (M = 480.9 g/mol) are of analytical grade and were used as received.

### 3.2. Synthesis of the Precursor Microparticles

The synthesis of P microparticles based on GMA, NVI and TEGDMA was carried out as described in a previous study [25] using the suspension polymerization technique. The suspension polymerization was carried out in a cylindrical reactor with a volume of 250 cm^3^ equipped with a mechanical stirrer, thermometer and reflux condenser. The reaction mixture consists of two phases: (1) aqueous phase consisting of 2 wt.% mixture of PVA and gelatin (70:30 g/g) and 3 wt.% inorganic salt (MgCl_2_·6H_2_O) and (2) organic phase consisting of GMA (40 moles), NVI (30 moles) and TEGDMA (30 moles), radical initiator (BOP) (2.5 wt.% with respect to the total amount of the monomers) and n–butyl acetate as a porogenic agent (D = 0.5, where D = mL n–butyl acetate/(mL n–butyl acetate +mL monomers)).

The organic/aqueous phase ratio was 1:9 (*v*/*v*) and the copolymerization reactions were conducted under a N_2_ atmosphere for 8 h at 78 °C and 1 h at 90 °C with a stirring speed of 300 rpm. Next, the P microparticles were separated by decantation, washed with hot water, dried at room temperature and extracted with methanol in a Soxhlet apparatus to remove the porogenic agent and traces of residual monomers.

### 3.3. Synthesis of Zwitterionic Microparticles

The zwitterionic microparticles (PB_1_, PB_2_ and PB_3_ microparticles) were prepared by the polymer analogous reaction described in our previous paper [26]. Thus, 3 g of P microparticles were swollen in 25 mL water for 24 h at room temperature. Then, the microparticles were brought into a round-bottom flask, over which a certain volume of 10% (*w*/*v*) betainizing agent solution (sodium monochloracetate or acrylic acid or methacrylic acid) was added. The amount of betainizing agent was calculated for a nitrogen/betainizing agent molar ratio of 1:2. The reaction mixture was kept under gentle stirring at 60 °C for 120 h. Afterwards, the zwitterionic microparticles were separated by filtration and washed with deionized water until the remaining unreacted reactants were removed. In the case of the reaction with sodium monochloroacetate, the purification of zwitterionic microparticles was carried out until the absence of Cl^−^ ions was achieved, with the washing waters being checked with a solution of AgNO_3_.

### 3.4. Experiments of the Sorption Studies

The sorption of DCH on precursor and zwitterionic microparticles was investigated in a batch system. The batch sorption was realized as follows: 10–100 mg of precursor or zwitterionic microparticles of known humidity placed in glass-stopped Erlenmeyer flasks were immersed in 10 mL aqueous solution of DCH of various concentrations (0.25 × 10^−3^–1 × 10^−3^ g/mL) at pH values ranging between 2 and 9. The initial pH of DCH solution was adjusted using 1 N HCl or 1 N NaOH. The Erlenmeyer flasks were placed in a thermostatic shaker bath (Memmert M00/M01, Memmert GmbH, Schwabach, Germany) under gentle stirring (180 rpm) for different contact times between 10 min and 360 min, at different temperatures (298, 303 and 308 K). After sorption, the microparticles were separated by centrifugation at 1000 rpm for 10 min. The concentration of the DCH in the supernatant solution before and after DCH sorption was determined by a UV-VIS Spectrophotometer (UV-VIS SPEKOL 1300, Analytik Jena, Jena, Germany) at a wavelength of 277 nm, using a calibration curve obtained by applying the Lambert–Beer law.

The quantities of DCH sorbed at any time (q_t_) and at equilibrium (q_e_) were calculated as follows:

(1) $$q_{t}=\frac{C_{0}-C_{t}}{w}\cdot V$$

(2) $$q_{e}=\frac{C_{0}-C_{e}}{w}\cdot V$$

where C_0_ is the initial concentration of DCH solution (g/mL); C_t_ is the concentration of DCH solution at any time (g/mL); C_e_ is the concentration of DCH solution at equilibrium (g/mL); V is the volume of DCH solution (L); and w is the amount of precursor or zwitterionic microparticles (g). Three independent measurements were performed for all the sorption data and the average values were taken into consideration.

### 3.5. Sorption Kinetic Experiments

In order to determine the mechanism of DCH sorption onto P, PB_1_, PB_2_ and PB_3_ microparticles, we processed the experimental data using four kinetic models, as follows: Lagergren model (pseudo-first-order kinetic model) [42], Ho model (pseudo-second-order kinetic model) [43], Elovich model [44] and Weber–Morris model (intra-particle diffusion model) [45]. The sorption kinetic studies were carried out for the concentration of DCH = 1 × 10^−3^ g/mL under optimum conditions (10 mg microparticles, pH = 8.5) at 308 K for 360 min with a stirring speed of 180 rpm. For the most accurate interpretation of the experimental data, in the case of the Lagergren, Ho and Elovich models, a nonlinear regression technique was used, while for the Weber–Morris model, the linear fitting technique was applied. Nonlinear regression is a more mathematically rigorous analysis that employs relevant computer software for parametric data estimation. The nonlinear regression method was carried out using OriginPro2023 software. To evaluate the best fitting of the models, two statistical error functions, namely correlation coefficient (R^2^) and chi-square test (χ^2^), were used [46]. The kinetic models describe the experimental data well when the values of R^2^ are close to unity and the values of χ^2^ are small. The four models mentioned above are described by the following equations:

Lagergren model:

(3) $$q_{t}=q_{e}\left(1-e^{-K_{1}t}\right)$$

Ho model:

(4) $$q_{t}=\frac{k_{2}q_{e}^{2}t}{1+k_{2}q_{e}t}$$

Elovich model:

(5) $$q_{t}=\frac{1}{\beta }ln(1+\alpha \cdot \beta \cdot t)$$

Weber–Morris model:

(6) $$q_{t}=k_{id}t^{0.5}+I_{d}$$

where q_t_ and q_e_ are the amount of DCH sorbed onto the microparticles at time t and at equilibrium (mg/g); k_1_ is the rate constant of the pseudo-first-order model (min^−1^); k_2_ is the rate constant of the pseudo-second-order model (g/mg·min); α is the initial sorption rate (mg/g·min); β is the desorption constant (g/mg); k_id_ is the intra-particle diffusion rate constant (mg/g·min); and I_d_ is the constant that gives information about the thickness of the boundary layer.

### 3.6. Sorption Isotherm Experiments

The equilibrium sorption studies of DCH on precursor and zwitterionic microparticles were performed at 298, 303 and 308 K and at different initial concentrations of DCH solution (0.25 × 10^−3^–1 × 10^−3^ g/mL). To describe the interactions between microparticles and DCH that occur in sorption processes, three model isotherms were used, namely Langmuir [47], Freundlich [48] and Dubinin–Radushkevich [49].

The nonlinear forms of the used isotherms can be written as follows:

Langmuir isotherm:

(7) $$q_{e}=\frac{q_{m}K_{L}C_{e}}{1+K_{L}C_{e}}$$

Freundlich isotherm:

(8) $$q_{e}=K_{F}C_{e}^{1/n_{F}}$$

Dubinin–Radushkevich isotherm:

(9) $$q_{e}=q_{D}\exp\left(-K_{D}\varepsilon_{D}^{2}\right)$$

where C_e_ is the DCH concentration at equilibrium (g/mL); q_m_ is the maximum sorption capacity (mg/g); K_L_ is the Langmuir constant (L/g), which represents the affinity between the sorbent and sorbate; K_F_ represents the sorption capacity for a unit equilibrium concentration (L/g); n_F_ is an empirical constant; q_D_ is the theoretical saturation capacity (mg/g); K_D_ (mol^2^/kJ^2^) is the constant which is related to the calculated average sorption energy E; and ε (kJ/mol) is the Polanyi potential. The constant K_D_ can give valuable information regarding the mean energy of sorption by the following equation: $E=(-2K_{D}{)}^{0.5}$.

The magnitude of the exponent 1/n_F_ gives information about the type of isotherm as follows: irreversible (1/n_F_ = 0), favorable (0 < 1/n_F_ < 1) or unfavorable (1/n_F_ > 1). Also, it is known that the sorption energy gives information about the type of sorption process: physical (1 kJ/mol < E < 8 kJ/mol), ion exchange (8 kJ/mol < E < kJ/mol) and chemical (E > kJ/mol) [50].

### 3.7. Thermodynamic Study

Temperature is one of the key parameters in the sorption process. The effect of temperature on the sorption of DCH onto precursor and zwitterionic microparticles was studied at 298, 303 and 308 K for 360 min. It is known that thermodynamic parameters—such as Gibbs free energy change ΔG, enthalpy change ΔH and entropy change ΔS—give us information about the mechanism and the type of sorption process. The value of ΔH and ΔS were calculated from the van’t Hoff equation [51]:

(10) $$lnK_{L}=\frac{∆S}{R}-\frac{∆H}{RT}$$

where K_L_ is the Langmuir equilibrium constant, R is the gas constant (8.314 J/mol·K) and T is the temperature (K).

The ΔS and ΔH values were calculated from the intercept and slope of the van’t Hoff plot of lnK versus 1/T. The values of ΔG were obtained using the following equation:

(11) ∆G=∆H−T∆S

### 3.8. In Vitro Drug Release Studies

In vitro drug release studies were realized by introducing the drug–microparticle systems (500 mg) into 50 mL of dissolution media with pH = 1.2 (simulated gastric solution) or pH = 7.4 (phosphate-buffered solution) at 37 °C, under gentle shaking (50 rpm) using a thermostated shaker bath. At predetermined time intervals, a known volume of the supernatant was collected, with syringes, from the dissolution medium. The amount of the released DCH at different periods of time was determined by UV-VIS spectrophotometry at 268 and 274 nm, respectively, using the calibration curves. Subsequently, the same volume of dissolution media was added into the release medium. The cumulative amount of DCH released (Q%) was calculated as follows:

(12) $$Q\left(\%\right)=\frac{10C_{n}+3\sum_{i=1}^{n-1}C_{i}}{M}\cdot 100$$

where M is the total mass of DCH sorbed onto precursor and zwitterionic microparticles; C_n_ and C_i_ are the concentration of DCH released from the precursor/zwitterionic microparticles loaded with DCH determined at different times.

The kinetic studies and the release mechanism from precursor and zwitterionic microparticles loaded with DCH were examined using the following mathematical models:

1. Higuchi model [52]:

(13) $$Q_{t}=K_{H}t^{0.5}$$

where Q_t_ is the amount of drug released at time t; K_H_ is the Higuchi dissociation constant.

2. Korsmeyer–Peppas model [53]:

(14) $$\frac{M_{t}}{M_{\infty }}=K_{r}t^{n}$$

where M_t_/M_∞_ is the fraction of drug released at time t; K_r_ is the release rate constant that is characteristic of drug–polymer interactions; n is the diffusion exponent corresponding to the release mechanism.

3. Baker–Lonsdale model [54]:

(15) $$\frac{3}{2}\left[1-{\left(1-F\right)}^{2/3}\right]-F=K_{BL}t$$

where F = M_t_/M_∞_; K_BL_ is the release constant.

4. Kopcha model [55]:

(16) $$M_{t}=At^{0.5}+Bt$$

where A is the diffusion constant and B is the erosion constant.

### 3.9. Characterization of Precursor and Zwitterionic Microparticles Loaded with DCH

The infrared spectra of the samples were recorded in Attenuated Total Reflectance (ATR-FTIR) configuration by means of a Bruker Vertex 70 spectrometer (Borken, Germany) equipped with a Specac^TM^ ATR module. The samples were scanned in the 4000–600 cm^−1^ spectral range, at 2 cm^−1^ resolution and by co-adding 128 scans.

The surface morphologies of the precursor/zwitterionic microparticles loaded with DCH were observed with a Verios G4 UC scanning electron microscope (Thermo Scientific, Brno, Czech Republic). The elemental composition was evaluated with an energy-dispersive X-ray spectroscopy detector (Octane Elect Super SDD detector, Ametek, Mahwah, NJ, USA). The microparticles were coated with 6 nm platinum using a Leica EM ACE 200 sputter coater (Leica microsystems, Wetzlar, Germany) to provide electrical conductivity and prevent charge buildup during exposure to the electron beam. SEM investigations were performed in high-vacuum mode using a concentric backscatter detector (CBS), working at an accelerating voltage of 5 kV.

## 4. Conclusions

In this study, we conducted a comprehensive investigation into the sorption and release of DCH from various precursor and zwitterionic microparticles. The optimal conditions for DCH loading onto precursor and zwitterionic microparticles were established by studying the influence of different parameters, such as dosage of microparticles, pH value, contact time, the initial concentration of DCH solution and temperature, respectively. From the sorption studies, it was found that the maximum sorption capacity was obtained in the case of the zwitterionic microparticles. The evaluation of the sorption performance of the precursor and zwitterionic microparticles was followed by kinetic, sorption and thermodynamic studies. The Ho and Elovich models indicate that chemisorption is the rate-controlling mechanism. The Langmuir and Dubinin–Radushkevich isotherms fit the experimental data, suggesting a higher affinity of zwitterionic microparticles for DCH compared to precursor ones, in agreement with the sorption capacity obtained from the experimental data. The thermodynamic study (positive values of ΔH and ΔS and negative values of ΔG) indicates that the sorption process is spontaneous and favorable. The analysis of the release data modeled by Higuchi, Korsmeyer–Peppas, Baker–Lonsdale and Kopcha models indicates that the release mechanism is predominantly based on diffusion.

## Figures and Tables

**Table 1:** The diameter, swelling degree in water and betainization degree values of precursor and zwitterionic microparticles.

Sample	Diameter (μm)	Swelling Degree in Water	Betainization Degree ^a^ (%)
P	215	285	-
PB_1_	231	389	90.20
PB_2_	234	366	84.24
PB_3_	235	343	80.98

^a^ the betainization degree was obtained from ATR-FTIR spectra [26].

**Table 2:** The experimental and determined kinetic model parameters for the sorption of DCH onto P, PB_1_, PB_2_ and PB_3_ microparticles.

	P	PB_1_	PB_2_	PB_3_
q_e,exp_ (mg/g)	100.500	157.860	150.400	144.100
*Lagergren model*				
q_e_ (mg/g) ^a^	94.960	148.779	142.18	136.109
k_1_ × 10^2^ (min^−1^) ^b^	3.024	3.984	3.767	3.330
R^2 c^	0.970	0.968	0.970	0.971
χ^2 d^	38.626	92.238	79.409	72.246
*Ho model*				
q_e_ (mg/g) ^a^	108.280	163.543	156.750	151.615
k_2_ × 10^4^ (g/mg·min) ^e^	3.385	3.247	3.175	2.812
R^2 c^	0.992	0.996	0.996	0.996
χ^2 d^	9.961	12.427	10.369	10.123
*Elovich model*				
α (mg/g·min) ^f^	7.593	22.864	19.550	14.577
β (g/mg) ^g^	0.044	0.034	0.035	0.035
R^2 c^	0.994	0.992	0.992	0.994
χ^2 d^	8.015	24.189	22.467	15.626

^a^ q_e_—the amount of DCH sorbed onto the microparticles at equilibrium; ^b^ k_1_—the rate constant of the pseudo-first-order model; ^c^ R^2^—correlation coefficient; ^d^ χ^2^—chi-square test; ^e^ k_2_—the rate constant of the pseudo-second-order model; ^f^ α—the initial sorption rate; ^g^ β—the desorption constant.

**Table 3:** Kinetic parameters based on the intra-particle diffusion equation for the sorption of DCH onto P, PB_1_, PB_2_ and PB_3_ microparticles.

	P	PB_1_	PB_2_	PB_3_
k_i,1_ (mg/g·min^1/2^) ^a^	9.179	14.588	14.052	13.388
I_1_ ^b^	2.584	13.015	10.043	5.972
R^2 c^	0.950	0.963	0.970	0.978
k_i,2_ (mg/g·min^1/2^) ^d^	2.461	2.675	2.525	2.783
I_2_ ^e^	59.845	109.782	104.995	94.257
R^2 c^	0.884	0.910	0.926	0.910

^a^ k_i1_ is the intra-particle diffusion rate constant for the first stage; ^b^ I_1_ is the constant for the first stage; ^c^ R^2^ is the correlation coefficient; ^d^ k_i2_ is the intra-particle diffusion rate constant for the second stage; ^e^ I_2_ is the constant for the second stage.

**Table 4:** Results of isotherm models for DCH sorption on the precursor microparticles.

P			
T (K)	298	303	308
*Langmuir model*			
q_m_ (mg/g) ^a^	99.81	101.67	103.44
K_L_ (L/g) ^b^	20.828	26.691	34.575
R^2 c^	0.999	0.998	0.996
χ^2 d^	0.070	0.097	0.117
*Freundlich model*			
K_F_ (L/g) ^e^	97.12	99.69	102.11
1/n_F_ ^f^	0.116	0.096	0.079
R^2 c^	0.930	0.926	0.966
χ^2 d^	3.991	3.196	1.083
*Dubinin-Radushkevich model*			
q_m_ (mg/g) ^a^	98.14	100.44	102.55
E (kJ/mol) ^g^	7.030	7.954	9.060
R^2 c^	0.998	0.996	0.998
χ^2 d^	0.116	0.166	0.054

^a^ q_m_—the maximum sorption capacity; ^b^ K_L_—the Langmuir constant; ^c^ R^2^—correlation coefficient; ^d^ χ^2^—chi-square test; ^e^ K_F_—the Freundlich constant; ^f^ n_F_—empirical constant; ^g^ E—sorption energy.

**Table 5:** Results of isotherm models for DCH sorption on the zwitterionic microparticles.

		PB_1_			PB_2_			PB_3_	
T (K)	298	303	308	298	303	308	298	303	308
*Langmuir model*									
q_m_ (mg/g) ^a^	142.81	153.27	160.48	139.50	150.96	154.29	138.14	143.58	148.17
K_L_ (L/g) ^b^	45.410	51.616	60.121	28.999	34.989	43.831	28.806	32.678	37.527
R^2 c^	0.997	0.996	0.996	0.995	0.996	0.997	0.995	0.995	0.997
χ^2 d^	0.167	0.246	0.243	0.475	0.430	0.224	0.468	0.464	0.288
*Freundlich model*									
K_F_ (L/g) ^e^	142.34	153.21	160.77	138.07	150.26	150.04	136.72	142.57	147.54
1/n_F_ ^f^	0.071	0.067	0.061	0.101	0.091	0.077	0.101	0.094	0.085
R^2 c^	0.955	0.953	0.953	0.948	0.959	0.947	0.963	0.962	0.957
χ^2 d^	2.857	3.239	3.210	5.404	4.398	4.703	3.792	3.779	4.054
*Dubinin-Radushkevich model*									
q_m_ (mg/g) ^a^	142.15	152.77	160.15	138.16	149.94	153.62	136.79	142.45	147.26
E (kJ/mol) ^g^	9.739	10.417	11.273	8.021	8.804	9.848	8.003	8.573	9.232
R^2 c^	0.999	0.998	0.999	0.997	0.998	0.999	0.997	0.998	0.999
χ^2 d^	0.019	0.090	0.052	0.279	0.165	0.046	0.272	0.231	0.052

^a^ q_m_—the maximum sorption capacity; ^b^ K_L_—the Langmuir constant; ^c^ R^2^—correlation coefficient; ^d^ χ^2^—chi-square test; ^e^ K_F_—the Freundlich constant; ^f^ n_F_—empirical constant; ^g^ E—sorption energy.

**Table 6:** The thermodynamic parameters for DCH sorption on the precursor and zwitterionic microparticles.

Sample Code	ΔH ^a^ (kJ/mol)	ΔS ^b^ (J/mol·K)	ΔG ^c^ (kJ/mol)			R^2 d^
			298 K	303 K	308 K	
P	38.668	154.976	−7.515	−8.290	−9.065	0.999
PB_1_	21.402	103.504	−9.442	−9.960	−10.477	0.993
PB_2_	31.503	133.650	−8.325	−8.993	−9.661	0.992
PB_3_	20.175	95.619	−8.320	−8.798	−9.276	0.997

^a^ ΔH—enthalpy change; ^b^ ΔS—entropy change; ^c^ ΔG—Gibbs free energy change; ^d^ R^2^—correlation coefficient.

**Table 7:** Kinetic parameters of drug release from P+DCH and PB_1_+DCH microparticles.

	P+DCH		PB_1_+DCH	
	pH = 1.2	pH = 7.4	pH = 1.2	pH = 7.4
*Higuchi Model*				
K_H_ (min^−0.5^) ^a^	4.482	4.360	3.848	3.895
R^2 b^	0.992	0.986	0.998	0.995
*Korsmeyer-Peppas Model*				
K_r_ (min^−0.5^) ^c^	0.069	0.081	0.041	0.048
n ^d^	0.426	0.410	0.496	0.476
R^2 b^	0.997	0.985	0993	0.994
*Baker-Lonsdale Model*				
K_BL_ (h^−0.5^) ^e^	0.028	0.031	0.020	0.023
R^2 b^	0.998	0.997	0.997	0.999
*Kopcha Model*				
A ^f^	6.103	6.869	6.452	7.275
B ^g^	−0.115	−0.143	−0.021	−0.052
A/B	53.070	48.035	307.238	139.904
R^2 b^	0.999	0.999	0.995	0.998

^a^ K_H_—the Higuchi dissociation constant; ^b^ R^2^—correlation coefficient; ^c^ K_r_—the Korsmeyer–Peppas release rate constant; ^d^ n—the diffusion exponent corresponding to the release mechanism; ^e^ K_BL_—the Baker–Lonsdale release constant; ^f^ A—the diffusion constant; ^g^ B—the erosion constant.

**Table 8:** Sorption capacity of different porous materials for DCH.

Sorbent	Sorption Capacity (mg/g)	Ref.
Magnetic porous silicas (Fe_3_O_4_@SiO_2_@mSiO_2_-CD)	200.000	[38]
Mesoporous silica	123.500	[39]
Mesoporous SiO_2_-ZnO composite	104.300	[40]
Magnetic Fe_3_O_4_@chitosan carbon microbeads	4.816	[41]
P	100.500	This work
PB_1_	157.860	This work

## Data Availability

Data are contained within the article.
